# Amyloid β inhibits retinoic acid synthesis exacerbating Alzheimer disease pathology which can be attenuated by an retinoic acid receptor α agonist

**DOI:** 10.1111/ejn.12142

**Published:** 2013-02-04

**Authors:** Maria B Goncalves, Earl Clarke, Carl Hobbs, Tony Malmqvist, Robert Deacon, Julian Jack, Jonathan P T Corcoran

**Affiliations:** 1 The Wolfson Centre For Age-Related Diseases, King's College LondonGuy's Campus, London, SE11UL, UK; 2 Department of Experimental Psychology, University of OxfordSouth Parks Road, Oxford OX1 3UD, UK

**Keywords:** Alzheimer's disease, amyloid β clearance, insulin-degrading enzyme, neprilysin, retinoic acid

## Abstract

The retinoic acid receptor (RAR) α system plays a key role in the adult brain, participating in the homeostatic control of synaptic plasticity, essential for memory function. Here we show that RARα signalling is down-regulated by amyloid beta (Aβ), which inhibits the synthesis of the endogenous ligand, retinoic acid (RA). This results in the counteraction of a variety of RARα-activated pathways that are key in the aetiopathology of Alzheimer's disease (AD) but which can be reversed by an RARα agonist. RARα signalling improves cognition in the Tg2576 mice, it has an anti-inflammatory effect and promotes Aβ clearance by increasing insulin degrading enzyme and neprilysin activity in both microglia and neurons. In addition, RARα signalling prevents tau phosphorylation. Therefore, stimulation of the RARα signalling pathway using a synthetic agonist, by both clearing Aβ and counteracting some of its toxic effects, offers therapeutic potential for the treatment of AD.

## Introduction

Alzheimer's disease (AD) has two major neuropathological hallmarks, the accumulation of amyloid beta (Aβ) in extracellular plaques and the presence of intracellular neurofibrillary tangles, whose main composition is hyperphosphorylated tau (Serrano-Pozo *et al*., 2011). The Aβ hypothesis (Hardy & Selkoe, 2002) about the cause of AD has prompted the search for treatments which clear the excess Aβ from the brain, as there is some evidence that the cause of late-onset AD is inadequate clearance, rather than excess production (Mawuenyega *et al*., 2010). Specific antibodies to Aβ have been a favoured approach (Delrieu *et al*., 2012), but recently a drug which penetrates the blood–brain barrier and elevates the level of apolipoprotein E (apoE), which acts as part of the physiological Aβ clearance mechanisms, has been suggested (Cramer *et al*., 2012). According to the most recent version of the Aβ hypothesis, the various pathogenic features of AD are primarily caused by soluble oligomeric forms of Aβ, particularly of its 42 amino-acid form, acting either extracellularly or intracellularly (LaFerla *et al*., 2007; Jin *et al*., 2011).

Microglia play a major role in Aβ clearance and they surround the Aβ plaques which induce a phagocytic response (Tuppo & Arias, 2005). They can also synthesize the Aβ-clearing enzymes insulin degrading enzyme (IDE) (Tuppo & Arias, 2005) and neprilysin (NEP) (Marr *et al*., 2004). However, inflammatory processes, caused by ‘activated’ microglia, are involved in the pathogenesis of AD (Tuppo & Arias, 2005; Rogers *et al*., 2007). One hallmark of this process is the secretion of the pro-inflammatory cytokine tumour necrosis factor (TNF) α, which may lead to further degeneration (Smith *et al*., 2012) and prevention of Aβ clearance (Hickman *et al*., 2008). In addition, the neurons themselves can remove the toxic oligomers of Aβ via the activation of IDE and NEP (Vekrellis *et al*., 2000; Carson & Turner, 2002). Therefore, an ideal therapeutic agent would be one that prevents the ‘activation’ of microglia as well as stimulating the IDE and NEP pathways in both microglia and neurons.

Although the aetiology of non-familial AD remains unclear, previous work has suggested that a deficit in retinoic acid (RA) signalling, which is essential for normal brain maintenance (Maden, 2007), may be associated with AD (Goodman & Pardee, 2003; Corcoran *et al*., 2004; Ding *et al*., 2008; Tippmann *et al*., 2009; Donmez *et al*., 2010; Jarvis *et al*., 2010). It has not yet been shown how a deficit in RA signalling could occur. Given the recently documented inverse correlation between intracellular calcium concentration and RA synthesis (Wang *et al*., 2011) and the role of Aβ in increasing intracellular calcium (Camandola & Mattson, 2011; Fedrizzi & Carafoli, 2011), we have investigated whether Aβ can prevent RA synthesis in the adult brain.

In earlier work, we showed that agonists of the retinoic acid receptor (RAR) α, but not of the RAR β or γ, were strikingly effective in lowering Aβ levels, having an especially strong action on intracellular Aβ42 (Jarvis *et al*., 2010). The mechanism was presumed to be explained by a reduction of Aβ production, due to the observed increase in activity of an alpha secretase, ADAM10, thus favouring the non-amyloidogenic processing of amyloid precursor protein (APP) over its amyloidogenic cleavage. However, given the dramatic decrease in extracellular soluble Aβ in Tg2576 mice as a response to RARα signalling, which does not alter APP processing (Jarvis *et al*., 2010), we have examined here whether there was an additional effect of RARα activity on the clearance of Aβ.

Our data suggest that Aβ can prevent the synthesis of RA in the adult brain, including the microglia. This is associated with the loss of activity of both NEP and IDE, an increase in the secretion of the inflammatory cytokine TNFα and the phosphorylation of tau. All of these processes can be reversed by an RARα agonist and the accumulation of Aβ in extracellular plaques reduced. In addition, we show that the cognitive behavioural deficits which develop in the Tg2576 mouse can be reversed.

## Materials and methods

### Animal treatments and cognitive behavioural studies

All procedures were performed in accordance with the Animal Scientific Procedures Act (1986), UK. Local ethics approval was obtained from King's College London in accordance with European Communities Council Directive of 24 November 1986. Tg2576 mice on a 129S6 background, APPSWE/tau(P301L) and respective wild-type (wt) littermate mice were purchased from Taconic Farms (Germantown, NY, USA). Mice were maintained on a 12/12-h light–dark cycle at 20–22 °C and given food and water *ad libitum*. Mice (*n* = 7–8 per group) were treated by intraperitoneal injections (i.p.) of either vehicle [80% dimethyl sulfoxide (DMSO) in distilled water] or 1 mg/kg with retinoids three times a week from 15 to 18 months of age for the Tg2576 mice and from 12 to 15 months age for the P301L mice. In addition the P301L mice were treated with 10 mg/kg of lithium (Sigma Aldrich, Dorset, UK) for the same amount of time as the retinoid treatment. These doses were based on previous work (Jarvis *et al*., 2010). The retinoids used were RARα selective, AM 580; RARβ selective, CD2019; or RARγ selective, CD437 (Jarvis *et al*., 2010). All the retinoid agonists were synthesized by Sygnature Chemical Services, Nottingham, UK.

### T-maze spontaneous alternation

This was carried out as previously described (Deacon & Rawlins, 2006) during the last 3 weeks of the retinoid treatment. Two trials were carried out separated by 30 min per group of mice (*n* = 7–8), and repeated on a weekly basis for a total of six trials. The mouse was placed at the end of the start arm facing away from the goal arms and allowed to freely explore the maze. Once the mouse tail tip was fully within one of the goal arms, the goal arm door was closed and the mouse allowed to explore for 30 s. The central partition and goal arm door were then removed and the mouse again placed in the start arm as above, and allowed free choice to enter either of the goal arms. Once the mouse tail tip was fully within one of the arms the trial was terminated and the correct number of alternations was recorded. If the mouse did not enter a goal arm after 90 s the trial was ended.

### Nest building

This was carried out as previously described (Deacon, 2006) in the last week of retinoid treatment. Mice (*n* = 7–8) were put in individual cages 2 h before dark. A nestlet (5 × 5 cm of cotton batting, Ancare; UK agent Lillico) was placed in the cage and the nest quality assessed the next day. The following scoring was used by an observer blinded to the treatment: (i) nestlet not noticeably touched; (ii) 50–90% nestlet intact; (iii) 50–90% nestlet shredded but no identifiable nest; (iv) 90% nestlet is torn and a flat identifiable nest and the walls higher than the mouse at less than 50% of its circumference; and (v) more than 90% nestlet is torn and the nest walls higher than the mouse for 50% of its circumference.

### Neuronal cultures

Cortical neurons were isolated from E15 mouse (NIH Swiss, Harlan, Oxon, UK). The brains were removed from the embryos and washed three times in PBS-1.5% glucose. Cortices without their meninges were then triturated through a 21G needle in the presence of PBS-1.5% glucose. The dissociated cells were then left for 5 min on ice to allow debris to settle. The supernatant was transferred to a 15 mL falcon tube and spun for 5 min at 134 ***g***. Cells were plated at a density 0.5 × 10^6^ per well on 13-mm^2^ glass coverslips in 24-well plates (Nunc, Invitrogen, Paisley, UK) precoated with 10 μg/mL poly-d-lysine (Sigma Aldrich, Dorset, UK). The neurons were grown in Neurobasal media (Invitrogen), supplemented with B-27 (Invitrogen), 2 mm glutamine, 1.5% glucose, 100 μg/mL streptomycin and 60 μg/mL penicillin (Invitrogen), at 37 °C in a humidified atmosphere of 95% air and 5% CO_2_. Cultures were used after 7 days and were greater than 98% neurons as judged by β-tubulin III staining. All the cultures used were similar in appearance at the time of treatment. The retinoids were made up at 1000 × stock concentrations in DMSO. Aβ42 (California Peptide Research Inc., Napa, CA, USA) was made up at 1000 × concentration in DMSO. Cortical neurons were cultured for 7 days in serum-free medium and then for 3 days with 1 μm human Aβ42 and either 0.1 μm of AM 580 alone or in the presence of Aβ. Culture conditions were three wells per treatment carried out three times.

### Microglia cultures

Primary mixed glial cultures were prepared as described previously (McCarthy & de Vellis, 1980) using a modified protocol. Briefly, mixed glial cultures were obtained from the cortices of postnatal mice (P5–P8). Cultures were maintained at 37 °C (5% CO_2_/95% O_2_) in medium containing 15% fetal bovine serum (Invitrogen) and 1% penicillin-streptomycin (Sigma Aldrich) for 10–14 days. Microglial cells were then harvested by forceful shaking for 1 min by hand and plated on poly-d-lysine-coated glass coverslips or plastic six-well plates. After 3 days, microglia cultures were treated for 3 days, with either vehicle or Aβ42 (0.02 μm), or AM 580 (0.1 μm) with or without Aβ42.

### Antibodies

The following antibodies were used: rabbit polyclonal anti-IDE (1 : 100, Abcam plc, Cambridge, UK), rabbit polyclonal anti-RARα (1 : 100, Santa Cruz Biotechnology, Heidelberg, Germany), mouse monoclonal anti-beta amyloid (1 : 100 6E10, Covance, Emeryville, CA, USA), anti-goat NEP (1 : 50, R&D Systems, Minneapolis, MN, USA), rabbit polyclonal anti-ionized calcium binding adaptor molecule 1 (Iba1, 1 : 1000, Wako Chemicals USA, Inc.), goat anti-Iba1 (1 : 200, Abcam), rabbit polyclonal anti-apoE [1 : 100 and 1 : 1000 (for immunohistochemistry and Western Blotting, respectively), Abcam). Secondary antibodies were AlexaFluor™ 594 and AlexaFluor™ 488 (1 : 1000, Molecular Probes, Invitrogen) and AlexaFluor™ 680 (1 : 5000, Molecular Probes, Invitrogen). DAPI was used to stain nuclei (1 μg/mL, Sigma Aldrich).

### Immunohistochemistry

#### Neuronal cultures

Immunohistochemistry was carried out as previously described (Goncalves *et al*., 2005). Neuronal cultures were washed with PBS for 1 min. They were then fixed in 4% paraformaldehyde for 30 min and washed three times for 5 min each in PBS-0.02% Tween. They were incubated in primary antibody in PBS-0.02% Tween at 4 °C overnight. Primary antibody was removed by washing three times for 5 min each in PBS-0.02% Tween. They were incubated in the secondary antibody for 1 h at room temperature in PBS-0.02% Tween, and then washed in PBS three times for 5 min. The coverslips were then mounted using FluroSave™ reagent (Merck, UK). Incubation of neurons with secondary antibodies in the absence of primary antibodies produced a very weak diffuse staining of cell bodies that did not overlap with the primary antibody-specific staining (data not shown).

#### Brain sections

Tg2576 and 129S2/SvHsd mice were deeply anaesthetized by i.p. injection of pentobarbitone, and transcardially perfused with heparinized saline. Brains were rapidly removed and longitudinally bidissected. Half hemispheres were post-fixed with 4% paraformaldehyde (in 0.1 m phosphate buffer) for at least 2 days at room temperature. Tissue was then embedded in paraffin wax and 5-μm sagittal sections cut throughout each block. Sets of consecutive sections, randomly chosen at four different levels per brain, were used for immunostainning. Sections were first dewaxed in xylene and 100% industrial methylated spirits, then heated in citric acid (10 mm, pH = 6), until boiling, then washed under a running tap for 5 min. Sections were then blocked with 1% BSA for 15 min, followed by overnight incubation at 4 °C with the primary antibody. For 6E10 staining, sections were incubated for 20 min in 70% formic acid before overnight incubation with primary antibody. After three 5-min washes with PBS-0.02% Tween, sections were incubated with the corresponding fluorescent secondary antibody. The slides were then mounted using FluroSave™ reagent (Merck).

### Microscopy

Multichannel fluorescence (DAPI–FITC–Texas Red filter set) images were captured using a Zeiss LSM 700 laser-scanning confocal microscope, with a 63 × oil-immersion Aprochromat objective (Carl Zeiss). Settings for gain, aperture, contrast and brightness were optimized initially, and held constant throughout each study so that all sections were digitized under the same conditions of illumination. Channels were imaged sequentially to eliminate bleed-through and multichannel image overlays were obtained using Adobe Photoshop 7.0 (Adobe Systems).

### F9-RARE LacZ reporter assay

Murine F9 embryonal carcinoma cells which stably express an RARβ2-promoter construct were used as previously described (Sonneveld *et al*., 1999). Cells were scored on a blue vs. not blue basis and number or percentage of LacZ-positive cells were quantified from five random fields of view from three independent experiments (Sonneveld *et al*., 1999).

### ELISAs and enzyme activity assays

Quantification of IDE activity was done using the Calbiochem® InnoZyme™ Insulysin/IDE Immunocapture Activity Assay Kit, following the manufacturer's instructions.

NEP activity assay was done following previous methods (Tamboli *et al*., 2010). NEP substrate was *N*-succinyl-Ala-Ala-Phe-7-amido-4-methylcoumarin (Sigma Aldrich) and NEP inhibitor thiorphan (Sigma Aldrich).

For TNFα and Aβ42 quantification, a mouse TNFα ELISA Kit (Molecular Probes, Invitrogen) and a human Aβ42 kit (Molecular Probes, Invitrogen) were used, respectively, following the manufacturers' instructions.

All results are a combination of three independent experiments and all samples have been normalized for protein concentration.

### Production of Aβ 42 oligomers

Aβ42 monomers and oligomers were prepared as previously described (Ryan *et al*., 2010). Briefly, human synthetic Aβ42 (California Peptide Research, Napa, CA, USA) was suspended in chilled 1,1,1,3,3,3 hexafluoro-2-propanol (HFIP; Sigma-Aldrich) to 1 mm. After vortexing, the Aβ/HFIP solution was aliquoted into microfuge tubes, lyophilized in a Speed-Vac and stored desiccated at −20 °C until required. Aβ oligomers were prepared by resuspending the desiccated peptide to 5 mm in anhydrous dimethyl sulfoxide (DMSO). Following a 10-min bath sonication, the suspension was diluted to 100 μm by cold PBS + 0.05% SDS, vortexed for 30 s and stored at 4 °C for 24 h to allow oligomer formation. The solution was then diluted to 50 μg/mL (11 μm) with PBS and added to neuronal cultures or incubated at 4 °C to allow further aggregation. Aβ monomers were prepared immediately before use. Desiccated peptide was resuspended to 5 mm in DMSO, as previously described, and further diluted to 50 μg/mL with PBS. The suspension was vortexed briefly and added to cell cultures.

### Quantification of Aβ oligomers in neuronal cultures

Amyloid beta oligomers were quantified in neuronal cultures as previously described (Cheng *et al*., 2009). Four images per culture condition were captured, and Aβ oligomers were quantified manually in five random fields, by an operator blinded to treatments. The experiment was repeated three times.

### Western blotting

Protein was isolated from the neuronal and microglial cultures in a lysis solution containing 20 mm Tris-HCl (pH 7.6), 1% NP-40 (Sigma Aldrich), 137 mm NaCl and 2 mm EDTA, with 1 mm phenylmethylsulfonyl fluoride, in the presence of a protease inhibitor mixture (Sigma Aldrich). Cell lysates were centrifuged for 10 min at 9000 ***g*** The protein concentration in the supernatants was determined with the BCA Protein Assay (Pierce, Rockford, IL, USA). Ten micrograms of protein was loaded onto 10% SDS-PAGE gels. Semi-dry blotting was performed, and the blots were probed with appropriate antibodies. The cell lysates were then incubated with the secondary antibody and visualized with an Odyssey infrared scanning system. For a loading control, the blots were probed with mouse anti-actin and developed as above. Signal density was calculated as the ratio of signal intensity to β-actin.

### Data analysis

Data were analysed using either one-way anova followed by Tukey's or Fisher's test, or for Tmaze one-way repeated-measures anova, followed by the Holm-Sidak test; or for nest building, one-way anova on ranks, followed by Dunn's test using sigma stat software (SPSS Software Ltd, Birmingham, UK). Comparisons were made between appropriate groups and differences were considered statistically significant at the level of *P* < 0.05. Results are given as mean ± SE or ± SD and *P*-values are provided as summary statistics.

## Results

### RARα signalling removes Aβ plaques and improves cognition in the Tg2576 mouse

**FIG. 1:**
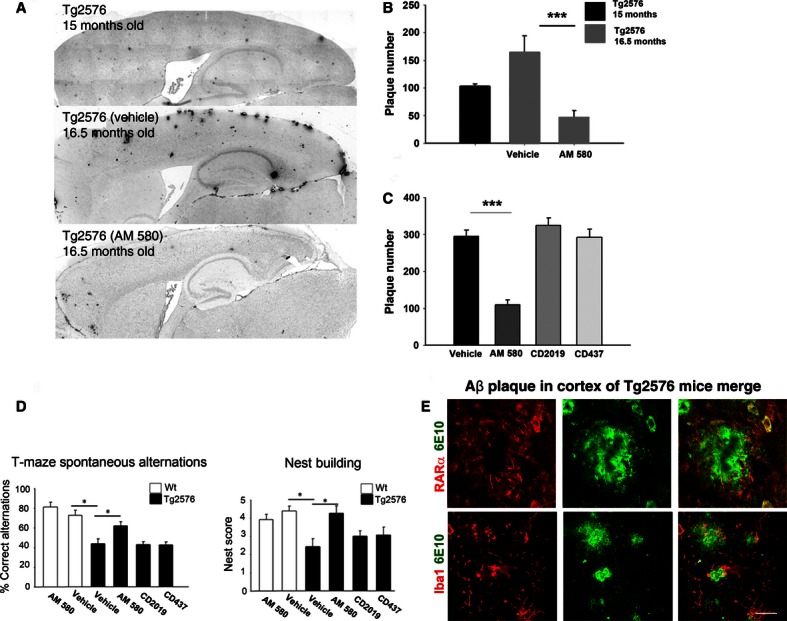
AM 580 induces Aβ plaque clearance and improves cognition in Tg2576 mice. (A) Immunohistochemistry for 6E10 in brains of Tg2576 mice. (B) Six weeks of AM 580 (1 mg/kg, i.p., three times a week) treatment significantly reduces plaque load in cortex and hippocampus in 15-month-old Tg 2576 mice (Student's, *t*-test, ***P < 0.001). (C) Twelve weeks of treatment with AM 580 (1 mg/kg, i.p., three times a week) significantly reduced plaque load whereas CD2019 or CD437 (both 1 mg/kg, i.p., three times a week) had no effect (one-way anova, followed by Tukey's test, ***P < 0.001). (D) Performance in behaviour tests of wt and Tg2576 mice treated either with vehicle or with retinoid agonists, T-maze spontaneous alternations and nest building, *n* = 7–8. For T-maze one-way repeated-measures anova, followed by Holm-Sidak test; *n* = 7–8, for nest building, one-way anova on ranks, followed by Dunn's test, *n* = 7–8, error bars are SEM, *P < 0.05. (E) RARα and microglia surround Aβ plaques in cortex of Tg2576 vehicle-treated mice. Scale bar = 20 μm.

Fifteen-month-old Tg2576 mice were treated with 1 mg/kg of either RAR α, β or γ selective agonists three times a week for 6–12 weeks and amyloid plaques were quantified in the cortex and hippocampus (Fig. 1A). In the 6-week treated mice, plaque load increased from 15 to 16.5 months by ∼60% in vehicle-treated mice. In contrast, treatment with the RARα agonist, AM 580, resulted in a net reduction by about 50% of the number of plaques 6 weeks earlier (Fig. 1B, one-way anova followed by Tukey's test, *F*_2,6_ = 10.109, *P* = 0.01). This result suggests that it is unlikely that the sole effect of the AM 580 is reduced production of Aβ and that it also facilitates clearance. Similarly, in animals treated for 12 weeks (Fig. 1C), the animals treated with AM 580 showed a significant decrease in plaque number compared with the vehicle-treated mice. Neither the RARβ agonist (CD2019) nor the RARγ agonist (CD437) had any effect on plaque load, matching our earlier result (Jarvis *et al*., 2010) that the reduction of Aβ levels is exclusively RARα−mediated (Fig. 1C, Tg2576 vehicle vs. Tg2576 AM 580, one-way anova followed by Tukey's test, *F*_3,44_ = 10.101, *P* < 0.001).

In parallel, the effect of each of the retinoid agonists on cognition was assessed by two tests, T-maze alternation and nest building (Deacon, 2006; Deacon & Rawlins, 2006). For these studies, age-matched wt littermates, treated with AM580, were included as another control group. The T-maze spontaneous alternation task was carried out during the last 3 weeks of retinoid treatment. The vehicle-treated Tg2576 mice made an average of 43% of successful trials compared with 72% for wt mice (Fig. 1D, one-way repeated-measures anova, *P* < 0.001 followed by Holm-Sidak test, *F*_3,16_ = 49.21, *P* < 0.001). In the RARβ- or γ-treated Tg2576 mice there was no significant difference in the number of correct alternations compared with the vehicle-treated Tg2576 ones (Fig. 1D, one-way repeated-measures anova as above all *P* > 0.05). However, in the RARα agonist-treated Tg2576 mice the number of correct alternations increased significantly compared with the vehicle-treated Tg2576 mice (Fig. 1D, one-way repeated-measures anova as above, *P* < 0.001) and was not significantly different from the vehicle-treated wt mice. In the nest building test, which was carried out in the last week of retinoid treatment, the vehicle-treated wt mice scored an average of 4.5/5, whilst the similarly treated Tg2576 mice scored 2.5/5 (Fig. 1D, one-way anova on ranks, followed by Dunn's test, *P* < 0.05). In the AM 580-treated Tg2576 mice there was a significant improvement in nest building compared with vehicle-treated Tg2576 (Fig. 1D, one-way anova on ranks as above, *P* < 0.05), which was comparable to the wt mice. By contrast, there was no improvement produced by the RARβ or γ agonist on nest building in the Tg2576 mice (Fig. 1D, one-way anova on ranks as above, all *P* > 0.05). Treatment with AM 580 in wt mice did not cause any significant difference in either test, compared with the wt vehicle-treated group (Fig. 1D).

### Aβ prevents RA synthesis

**FIG. 2:**
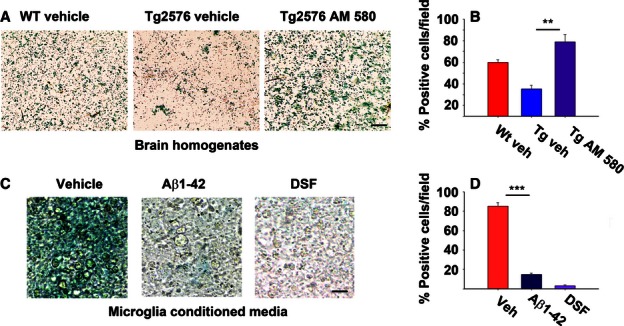
AM 580 attenuates Aβ-induced decrease of RA synthesis. (A) X-gal staining of F9 cells showing RA synthesis in brain homogenates; scale bar = 150 μm. (B) Quantification of RA synthesis shows that AM 580 (1 mg/kg, i.p., three times a week for 3 months) completely restores Aβ-induced decrease in RA synthesis in Tg2576 mice brains (one-way anova, followed by Tukey's test, **P < 0.01). (C) X-gal staining in F9 cells showing RA synthesis from microglia cultures; scale bar = 50 μm. (D) Quantification of positive cells shows that Aβ1-42 (0.02 μm, 3 days) significantly blocks RA synthesis in microglia cultures (one-way anova, followed by Tukey's test, ***P < 0.001); *n* = 3, for all experiments. Error bars are SEM.

Microglial cells play an essential role in Aβ clearance (Cameron & Landreth, 2010), so we investigated whether the RARα was expressed in microglia present in the vicinity of amyloid plaques. Immunostaining with the microglia marker Iba1 and with RARα antibody, in cortices of Tg2576 mice, showed co-localization (data not shown) and both were found to be expressed around amyloid plaques, which were labelled with the marker 6E10 (Fig. 1E). As there is a failure of plaque clearance in the Tg2576 mice, despite the presence of some RARα, we investigated whether it was a reduction in the production of the endogenous ligand, RA, or a problem downstream of the retinoid activation, which was responsible. To make a direct check on the synthesis of RA, we used a bioassay with a RARE-lacZ RA reporter cell line, F9 (Sonneveld *et al*., 1999). F9 cells were treated with homogenates of whole brain hemispheres of either wt, vehicle-treated Tg2576 or AM 580-treated Tg2576 mice. After overnight incubation, F9 cells were stained for X-gal and the intensity of staining was used to quantify RA synthesis. There was a significant decrease in RA synthesis in vehicle-treated Tg2576 mice, as shown by the lack of positive blue cells compared with wt mice, and this was reversed by AM 580 treatment (Fig. 2A and B, one-way anova followed by Tukey's test, *F*_2,6_ = 21.295, *P* = 0.002). To confirm that this effect was at least partly taking place in the microglia, F9 cells were treated with conditioned media taken from microglial cultures that were treated for 3 days with either vehicle, Aβ42 or Disulfiram (DSF), which blocks RA synthesis (Lipsky *et al*., 1997). After 1 day F9 cells were stained for X-gal, as above. Microglia were shown to synthesize RA, which was prevented in the presence of DSF or Aβ42 (Fig. 2C and D, one-way anova followed by Tukey's test, *F*_2,6_ = 371.545, *P* < 0.001).

### RARα signalling stimulates Aβ clearance by increasing NEP and IDE activity

**FIG. 3:**
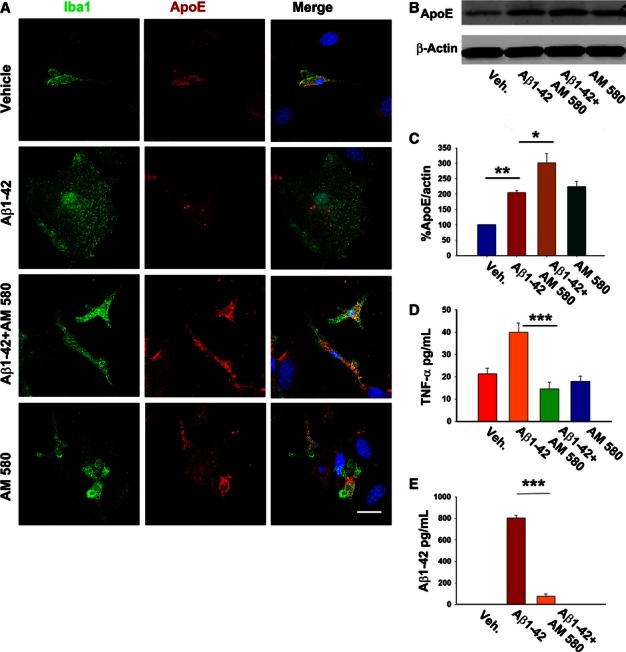
AM 580 induces Aβ clearance in microglia via up-regulation of apoE and down-regulation of TNFα. (A) Expression of apoE in microglia cultures; scale bar = 50 μm. (B, C) Quantification of microglial apoE by Western blotting (one-way anova, followed by Tukey's test, **P < 0.01, *P < 0.05). (D) Suppression of TNFα secretion by AM 580 (0.1 μm, 3 days) in Aβ- (0.02 μm, 3 days) treated microglia cultures measured by ELISA (one-way anova, followed by Tukey's test, ***P < 0.001). (E) AM 580 (0.1 μm, 3 days) induces clearance of Aβ1-42 from media in Aβ1-42- (0.02 μm, 3 days) treated microglia cultures (Student's, *t*-test, ***P < 0.001).

We next looked at the effect of RARα signalling on apoE expression, as it is well known that increased Aβ in the brain produces a corresponding increase in apoE (Rossello *et al*., 2012), perhaps by direct transcriptional action (Maloney & Lahiri, 2011), but yet not sufficient to induce effective clearance of Aβ (Cramer *et al*., 2012). We confirmed that Aβ increases apoE in microglia cultures, and more importantly that when AM 580 was concomitantly added to the cultures, it significantly induced apoE expression compared with Aβ alone (Fig. 3A–C, one-way anova followed by Tukey's test, *F*_3,8_ = 33.151, *P* = 0.03). We then approached downstream pathways involved in Aβ clearance.

It is well known that early microglial recruitment to the amyloid plaques promotes their clearance (Hickman *et al*., 2008), but the subsequent release of inflammatory cytokines, including TNFα, leads to the downregulation of genes which are involved in the clearance of Aβ, such as IDE and NEP (Nalivaeva *et al*., 2012). Therefore, we checked whether the RARα pathway had an anti-inflammatory action by downregulating TNFα secretion. Microglial cultures were treated with either vehicle or 20 nm Aβ42, in the presence or absence of AM 580, for 3 days. TNFα was then quantified in the media using a specific TNFα ELISA kit. We found that AM 580 significantly decreased TNFα release from Aβ-treated microglial cultures (Fig. 3D, one-way anova followed by Tukey's test, *F*_3,8_ = 14.446, *P* < 0.001). To test the capability of RARα activation to induce Aβ clearance in Aβ42-treated microglial cultures, the amount of Aβ42 in the medium was assayed using a specific human Aβ42 ELISA kit. AM 580 significantly reduced the level of Aβ in the medium, by about 90%, compared with control cultures (Fig. 3E, Student's *t*-test, *t*_10_ = 7.444, *P* < 0.001). These results, taken together, clearly illustrate the connection between RARα signalling, modulation of TNFα production and Aβ clearance by the microglia.

**FIG. 4:**
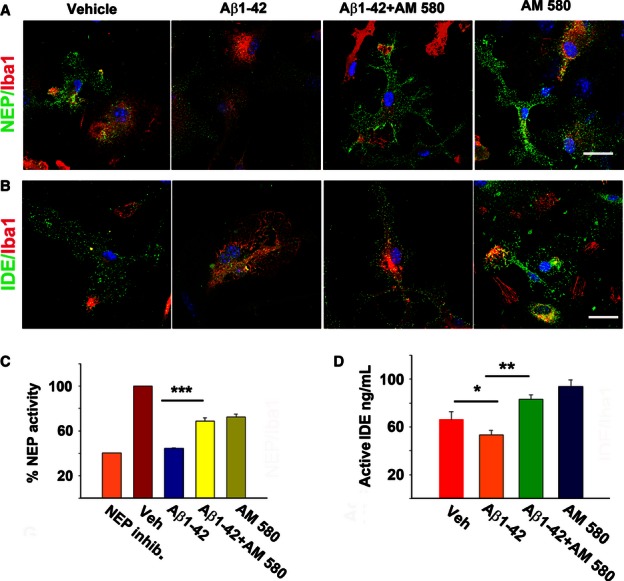
AM 580 induces Aβ clearance in microglia by increasing activity of NEP and IDE. Expression of NEP (A) and IDE (B) in microglia cultures; scale bar = 20 μm. (C) NEP activity in microglia cultures is significantly higher in Aβ1-42-treated cultures treated concomitantly with AM 580 compared with Aβ1-42-treated (one-way anova, followed by Tukey's test, ***P < 0.001). (D) IDE activity significantly increases in Aβ1-42-treated microglia cultures with concomitant treatment of AM 580 compared with Aβ1-42-treated (one-way anova, followed by Fisher's test, *P < 0.05, **P < 0.01). *n* = 3 for all experiments, Error bars are SEM.

Two of the major mechanisms by which microglia promote proteolytic Aβ clearance are through the action of NEP and IDE, with NEP being the sole documented mechanism for cleaving oligomeric Aβ (Nalivaeva *et al*., 2012). The actions of these two enzymes are facilitated by the presence of apoE (Jiang *et al*., 2008). We therefore checked the activity and location of these two enzymes in Aβ42-treated microglia, in the presence or absence of AM 580. Microglial cultures were treated as before, and double stained for both Iba1 and either NEP or IDE. Both proteolytic enzymes showed a decrease in expression in Aβ-treated microglia compared with vehicle-treated microglia, which was reversed by AM 580 treatment (Fig. 4A and B). Both enzymes were found to be expressed on the microglial surface. As NEP acts as an ectoenzyme (Marr *et al*., 2004), to test cell surface activity we used an NEP activity assay (Tamboli *et al*., 2010), whereas IDE activity was measured in the culture medium, as microglial cells release substantial amounts of IDE (Qiu *et al*., 1997). We found that Aβ caused a significant decrease in IDE activity and AM 580 reversed the Aβ-induced suppression of activity, to above vehicle levels for IDE and near to vehicle levels for NEP (Fig. 4C and D, for NEP, one-way anova followed by Tukey's test, *F*_2,5_ = 97.066, *P* < 0.001; for IDE, one-way anova followed by Fisher's test, *F*_3,9_ = 12.846, *P =* 0.04 for veh. vs. Aβ and *P* = 0.01 for Aβ vs. AM 580-treated cultures).

### RARα signalling removes Aβ oligomers

**FIG. 5:**
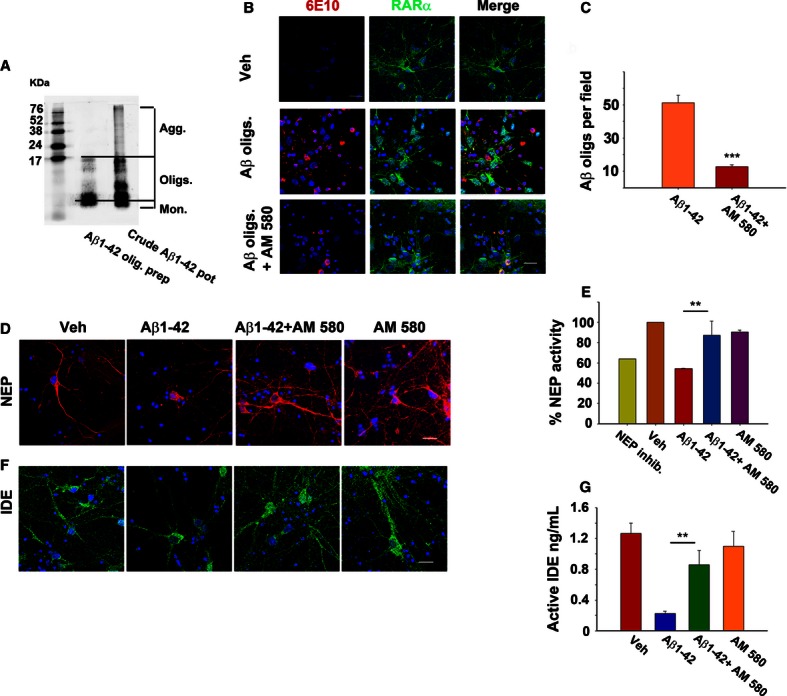
AM 580 induces Aβ oligomeric clearance in neuronal cultures by increasing NEP and IDE activity. (A) Western blots of Aβ protein using 6E10 antibody, illustrating the formation of oligomeric forms of Aβ. (B) Immunostaining showing Aβ1-42 oligomers and RARα in cortical neuronal cultures; scale bar = 30 μm. (C) Quantification of oligomers shows significant decrease in the presence of AM 580 (Student's *t*-test, ***P < 0.001). (D) Expression of NEP in neuronal cultures; scale bar = 20 μm. (E) NEP activity in membrane lysates is significantly increased in Aβ1-42- (1 μm, 3 days) treated cultures treated concomitantly with AM 580 (0.1 μm, 3 days) compared with Aβ1-42-treated (one-way anova, followed by Fisher's test, **P = 0.001). (F) Expression of IDE in neuronal cultures; scale bar = 20 μm. (G) Active IDE in neurons is significantly increased in Aβ1-42-treated cultures treated concomitantly with AM 580 compared with Aβ1-42-treated (one-way anova, followed by Fisher's test, **P = 0.002).

The degradation of Aβ plaques by cleaving enzymes could lead to a local increase in soluble oligomeric Aβ. Indeed, there is a local association between plaques and raised concentration of potentially neurotoxic Aβ dimers (Larson & Lesne, 2012). Therefore, we asked if neuronal RARα signalling could help clear Aβ oligomers. Cortical neurons were cultured for 3 days with Aβ42 oligomers (Fig. 5A), with or without AM 580. Immunostaining with 6E10 and RARα showed that in Aβ42 oligomer-treated cultures, abundant oligomers of Aβ surrounded the neuronal surface, whereas in the presence of AM 580 significantly fewer oligomers were seen (Fig. 5B and C, Student's *t* test, *t*_6_ = 8.311, *P* < 0.001). This suggests that AM 580 induces Aβ clearance mechanisms in neurons. To confirm this, we first immunostained for NEP and IDE in the cultured neurons. This showed a cytoplasmic/perinuclear distribution in Aβ-treated cultures (Fig. 5D and F) but the enzymes became membrane-distributed upon treatment with AM 580, as in the vehicle-treated neurons. The major neuronal site for the degrading activity of both these enzymes is the cell surface (Vekrellis *et al*., 2000; Leissring *et al*., 2003). We assayed for NEP activity as before, and for IDE activity, using neuronal membrane lysates. AM 580 significantly increased both enzymes' activity, reversing the Aβ-induced suppression (Fig. 5E and G, for NEP, one-way anova followed by Fisher's test, *F*_3,8_ = 7.835, *P* = 0.001; for IDE, one-way anova followed by Fisher's test, *F*_3,8_ = 9.3, *P* = 0.002).

### RARα signalling inhibits tau phosphorylation

**FIG. 6:**
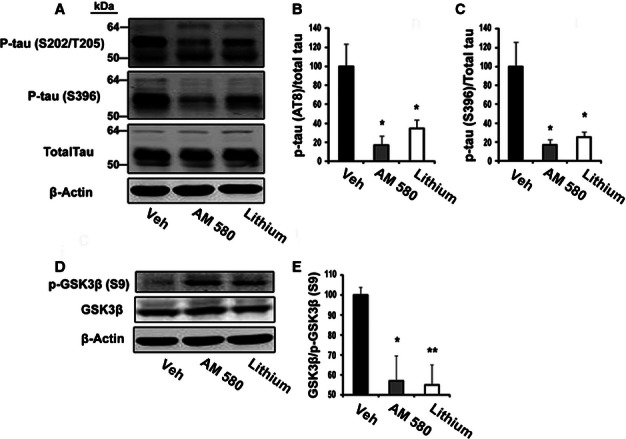
AM 580 prevents tau phosphorylation in APPSWE/tau(P301L) mice. (A) Western blots of phosphorylated tau at epitopes Ser202/Thr205 (AT8) and Ser396 (S396) and total tau. (B) Quantitative analysis of protein levels shows a marked decrease in phosphorylation was observed at the AT8 (one-way anova followed by Tukey's test, *P < 0.01). (C) S396 epitope of tau at 54 kDa in AM 580 and lithium compared with vehicle-treated mice. (D) Representative Western blots of phosphorylated GSK3β at Ser9 [*P*-GSK3β (Ser9)] and total GSK3β. (E) Quantitative analysis of protein levels revealed increased levels of GSK3β phosphorylated at the inhibitory epitope Ser9, as demonstrated by a decrease in the GSK3β/*P*-GSK3β (Ser9) ratio in AM 580 and lithium compared with vehicle-treated mice (one-way anova followed by Tukey's test, *P < 0.05, **P < 0.01). Data are mean values expressed as a percentage of vehicle (set to 100%); *n* = 3 for all experiments. Error bars are SEM.

A second hallmark of AD pathology is the abnormal hyperphosphorylation and intracellular accumulation of the microtubule-associated protein, tau, into neurofibrillary tangles. It is still unresolved in what ways the phosphorylation of tau contributes to toxicity, but recent work suggests that soluble intracellular oligomers, particularly dimers, of Aβ lead to phosphorylation of tau (Jin *et al*., 2011) freeing it from the microtubule network and in consequence impairing some forms of intracellular transport, including that of mitochondria and some cargo vesicles (Ittner *et al*., 2009; Kopeikina *et al*., 2011). Thus, it is an important element of AD therapeutics that tau phosphorylation is inhibited. We therefore investigated the effect of AM 580 on tau phosphorylation. APPSWE/tau(P301L) transgenic mice were treated with vehicle, AM 580 or lithium for 3 months and their homogenized cortices were analysed by Western blotting. In the presence of AM 580 or lithium, phosphorylation was reduced at the AT8 and s396 (PHF-1) epitopes, compared with the vehicle-treated mice (Fig. 6A–C, for AT8, Student's *t*-test all against vehicle, *t*_9_ = 3.274, vehicle vs. AM 580, *P* = 0.01, vehicle vs. lithium, *P* = 0.01; for s396, Student's *t*-test all against vehicle, *t*_9_ = 3.041, vehicle vs. AM 580, *P* = 0.014; vehicle vs. lithium, *P* = 0.013). This was associated with an increased expression of phosphorylated GSK3beta at the serine 9 residue (Fig. 6D and E, Student's *t*-test all against vehicle, *t*_10_ = 3.327, vehicle vs. AM 580, *P* = 0.008; vehicle vs. lithium, *P* = 0.002).

## Discussion

Retinoic acid receptor α is the predominant RAR expressed in the adult central nervous system (Corcoran *et al*., 2004; Agudo *et al*., 2010; Meng *et al*., 2011) and it is becoming increasingly clear that it plays an important role in the maintenance of brain homeostasis (Lane & Bailey, 2005; Luo *et al*., 2009; Duong & Rochette-Egly, 2011). We have shown here that RARα signalling modulates key physiological mechanisms involved in Aβ clearance and how this signalling system is compromised by Aβ, which reduces the synthesis of the endogenous ligand RA. As RA and the RARα system have been implicated as one mechanism contributing to homeostatic synaptic plasticity (Aoto *et al*., 2008; Chen *et al*., 2008; Maghsoodi *et al*., 2008; Sarti *et al*., 2012), the question should be raised as to whether Aβ also participates in this regulatory mechanism, given that normally synaptic activity sets its extracellular level (Cirrito *et al*., 2005). There is evidence that endogenous Aβ has a number of physiological roles, including neuronal survival (Plant *et al*., 2003), modifying the expression of potassium channels (Plant *et al*., 2006) and of the probability of transmitter release (Abramov *et al*., 2009) as well as being necessary for synaptic plasticity and memory (Puzzo *et al*., 2008, 2011); most of these effects may be achieved by the monomeric form of Aβ.

The evidence for Aβ depression of RA production comes first from *in vivo* experiments showing this result for Tg2576 mouse brains. We do not know the concentration of Aβ reached in these brains, but administration of the RARα agonist restores RA levels back to normal, wt, levels, compared with a ∼50% reduction in the Tg2576 mice. We decided to explore microglial RA synthesis, as these cells are abundant in the AD brain (Hickman *et al*., 2008) and play a role in the pathology of AD (Rogers *et al*., 2002). In cultured microglia cells, RA synthesis was repressed in response to Aβ, cells became activated and significantly increased TNFα secretion. Co-treatment with an RARα agonist reversed all these events and also correlated with an increase in apoE expression.

We have previously reported that in RA-deficient rats there is a reduction in the expression of RARα (Corcoran *et al*., 2004) and we show here that synthesis of RA is compromised in the cortices of Tg2576 mice. Thus, Aβ reduces the levels of both the agonist and the receptor and a good way to restore the level of RARα is to administer an RARα agonist. There is evidence that the same pathophysiology occurs in the AD brain, as we have reported that there is an approximately 30% reduction in RARα levels compared with age-matched normal brains (Corcoran *et al*., 2004).

Recent work has shown that retinoic acid-X receptor (RXR) ligands may have potential therapeutic in AD (Cramer *et al*., 2012). However, to date no endogenous ligand of the RXR have been identified. Whilst 9-*cis* RA can activate RXRs this is very difficult to detect in animal tissues; alternative ligands such as unsaturaturated fatty acids have been shown to bind but with very low affinity (Wolf, 2006). Thus, activating a pathway that is not deficient in AD may lead to effects unrelated to AD. Here we have shown that the RA signalling pathway can be directly linked to Aβ, accumulation of which compromises RARα signalling, which in turn can be restored by a specific RARα agonist.

We have shown here that the RARα signalling system upregulates several Aβ clearance mechanisms, in both neurons and microglia, including the important NEP proteolytic pathway, which is the only known mechanism for cleaving oligomeric amyloid (Nalivaeva *et al*., 2012). We have also provided evidence that this retinoid system counteracts other potentially toxic effects of Aβ, such as impairment of cognitive behaviour, the release of a pro-inflammatory cytokine (TNFα) from microglia and the phosphorylation of tau. These observations should be put together with our earlier demonstration that activation of the RARα pathway reduces the amyloidogenic production of Aβ and also acts neuroprotectively, reducing the Aβ-induced neuronal cell death in cortical cultures (Jarvis *et al*., 2010).

We have shown previously that RARα signalling will increase the activity of the non-amyloidogenic pathway and hence shed sAPPα into the extracellular medium. Amongst the actions of this molecule is amelioration of the calcium dyshomeostasis produced by Aβ (Mattson *et al*., 1993) and the phosphorylation of GSK-3β at the serine 9 site (Jimenez *et al*., 2011). On the other hand, given the large number of neuronal genes that are potentially regulated by the retinoids (Lane & Bailey, 2005), it seems likely that other RARα pathways are activated. This possibility then prompts the question about how extensive is the total list of effects produced by activating the RARα pathway and, in particular, whether they would all be beneficial in the treatment of AD. Given that our evidence suggests that all the effects of the RARα system will be equally down-regulated by Aβ in AD because it inhibits the synthesis of the endogenous agonist, RA, it would therefore be expected that all the effects would be a return towards normality.

An ideal therapeutic intervention would be to use an RARα agonist at a dose which restores the RARα to its normal level, giving this homeostatic mechanism in the brain an opportunity to reduce the production, enhance the clearance and counteract the untoward effects of excess Aβ, including the inhibition of the local synthesis of the endogenous ligand, RA. We regard the development of safe RARα agonists, therefore, as a promising approach to arresting the pathophysiology of AD.

## Abbreviations

Aβ: amyloid beta
AD: Alzheimer's disease
apoE: apolipoprotein E
APP: amyloid precursor protein
DMSO: dimethyl sulfoxide
DSF: disulfiram
HFIP: 1,1,1,3,3,3 hexafluoro-2-propanol
IDE: insulin degrading enzyme
i.p: intraperitoneal
NEP: neprilysin
RA: retinoic acid
TNF: tumour necrosis factor
wt: wild-type
